# Effects of Dietary Colostrum Basic Protein on Bone Growth and Calcium Absorption in Mice

**DOI:** 10.3390/nu16050664

**Published:** 2024-02-27

**Authors:** Yiran Zhang, Ziyu Qiao, Jiale Yu, Chenhong Shi, Rui Quan, Wen Zhang, Ran Bi, Hongliang Li, Wentao Qian, Menghui Wang, Yixuan Li

**Affiliations:** 1 Key Laboratory of Precision Nutrition and Food Quality, Department of Nutrition and Health, China Agricultural University, Beijing 100083, China; yiran1119@cau.edu.cn (Y.Z.); qiaoziyu@cau.edu.cn (Z.Q.); yujiale@cau.edu.cn (J.Y.); shichenhong@cau.edu.cn (C.S.); ruiquan@cau.edu.cn (R.Q.); zwzn@cau.edu.cn (W.Z.); biran@cau.edu.cn (R.B.); 2 Mengniu Hi-Tech Dairy Products (Beijing) Co., Ltd., Beijing 101100, China; lihongliang@mengniu.cn; 3 Inner Mongolia Mengniu Dairy (Group) Co., Ltd., Hohhot 011500, China; qianwentao@mengniu.cn (W.Q.); wangmenghui@mengniu.cn (M.W.)

**Keywords:** bovine colostrum basic protein, bone growth, bone density, calcium metabolism, calcium absorption

## Abstract

Colostrum basic protein (CBP) is a trace protein extracted from bovine colostrum. Previous studies have shown that CBP can promote bone cell differentiation and increase bone density. However, the mechanism by which CBP promotes bone activity remains unclear. This study investigated the mechanism of the effect of CBP on bone growth in mice following dietary supplementation of CBP at doses that included 0.015%, 0.15%, 1.5%, and 5%. Compared with mice fed a normal diet, feeding 5% CBP significantly enhanced bone rigidity and improved the microstructure of bone trabeculae. Five-percent CBP intake triggered significant positive regulation of calcium metabolism in the direction of bone calcium accumulation. The expression levels of paracellular calcium transport proteins CLDN2 and CLDN12 were upregulated nearly 1.5-fold by 5% CBP. We conclude that CBP promotes calcium absorption in mice by upregulating the expression of the calcium-transporting paracellular proteins CLND2 and CLND12, thereby increasing bone density and promoting bone growth. Overall, CBP contributes to bone growth by affecting calcium metabolism.

## 1. Introduction

Bone has important physiological functions such as protecting the vital organs of the body, storing minerals, and providing a hematopoietic environment [1,2]. Any pathology of bone may damage one or more bodily functions [3]. The bone formation and absorption cycle maintains bone health during bone development and throughout the life of the animal [4,5]. The balance is regulated by hormones, vitamins, growth factors, and cytokines [6], especially the trace element metabolism of the body [7]. In children and adolescents, for the accumulation of bone mass, bone formation is greater than bone resorption, but the opposite is true in adults (especially in old age), when bone loss is faster. Therefore, the amount of bone mass accumulated in the early stage of life is a determining factor of the level of bone mass in the later years and the occurrence of fractures due to bone fragility.

Optimizing calcium and protein intake during the growth process increases bone formation in the early stage of bone development so that the body can obtain the best peak bone mass and strength. As the main inorganic component of bone, calcium plays an irreplaceable role in the human body [8,9]; 99% of the body’s calcium is stored in the bones in the form of hydroxyapatite, which contributes to their strength. The body’s serum calcium concentration is usually stable, and fluctuation triggers the regulation of calcium metabolism with the deposition or release of bone calcium and the absorption of calcium [10,11]. In addition, in the process of maintaining the stability of serum calcium, parathyroid hormone (PTH), 1,25(OH)2D3 (the active form of vitamin D3), and calcitonin (CT) maintain calcium homeostasis by acting on the bone, kidney, and intestine [12,13]. PTH mobilizes bone calcium into the blood when serum calcium is decreased and activates 1,25(OH)2D3 to promote intestinal calcium absorption. When serum calcium is increased, CT is released to reduce serum calcium by increasing the excretion of ionic calcium by the kidney, which promotes bone calcium deposition and inhibits its absorption in the intestine.

Bovine colostrum basic protein (CBP) is a milky white powder containing a large amount of protein that is obtained from bovine colostrum by sterilization, degreasing, centrifugation, and removal of casein, α-lactoprotein, and β-lactoglobulin. The composition of CBP is shown in Table 1. It is believed that proteins with molecular weights of 1–30 kDa are the key components of CBP in promoting bone activity [14]. However, due to the complex and diverse protein components of CBP, the biological activity of specific protein types has not been analyzed. Interest in the use of CBP to improve bone health is growing because CBP appears to be a potent bone-stimulating factor. CBP potently promoted the bone mineral density (BMD) of rats. In vivo experiments showed that it increased the content of serum osteoblastic markers, indicating that CBP can regulate bone metabolism and promote bone growth [14]. However, the maintenance of bone growth and health is also affected by the body’s mineral metabolism [15]. Therefore, this study seeks to explore whether dietary CBP can affect bone development by regulating calcium metabolism.

## 2. Materials and Methods

### 2.1. Animals and Experimental Design

In a controlled environment (12-h light/dark cycle; temperature: 22 ± 1 °C), 100 male C57BL/6JN mice (Beijing Vital River Laboratory Animal Technology) aged 4 week were randomly assigned (*n* = 20 mice per group) to 5 groups: normal control (NC), 0.015% CBP, 0.15% CBP, 1.5% CBP, and 5% CBP. The composition of both diets is shown in Table 2. Apart from protein composition, all the other constituents were identical between these 5 diets. All animals were obtained from Weitong Lihua Laboratory Animal Technology Co., Ltd. (Beijing, China).

After 4 week, mice (*n* = 3) were placed in a special mouse metabolic cage that could separate feces and urine through a funnel at the bottom; the feed intake, urine volume, and fecal volume of mice were recorded within 24-h. A single mouse’s feces should be dried in a drying oven to a constant weight before being weighed and recorded. Collected urine and dried feces were stored at −20 °C for the detection of calcium content. Mice (*n* = 12) were necropsied, and femur and partial small intestinal segments were excised. The left femur (*n* = 6) was fixed with 4% paraformaldehyde for microscopic CT analysis. The right part of the femur from the same mouse (*n* = 6) was wrapped with gauze impregnated with normal saline and stored at −20 °C for bone biomechanical examination. The left tibia (*n* = 6) from the same mouse was prepared for bone mineral detection. The remaining left femur (*n* = 6) was for tissue staining, and the remaining right femur (*n* = 6) was dried in the oven to estimate the weight, length, and diameter of the femur. After centrifuging at 3000× *g* for 15 min at 4 °C, serum (*n* = 6) was obtained and stored at −80 °C for calcium (105-000453-00, Mindray, Shenzhen, China), phosphorus (105-015568-00, Mindray, Shenzhen, China), parathyroid hormone (JN19040, Jining Shiye, Shanghai, China), and calcitonin concentration (JN19883, Jining Shiye, Shanghai, China). A portion of the jejunum and ileum were flash-frozen in liquid nitrogen for later protein extraction.

### 2.2. Body Composition Analysis

Each mouse was weighed and then detected with a sober animal body composition analyzer (QMR, Niumag Corporation, Suzhou, China) for the content and proportion of fat and muscle in the body.

### 2.3. Motor Ability Test

A motor ability test was performed by the motorized treadmill (ZS-PT-III, Zhongshi Technology, Beijng, China). After 3 days of acclimation, the mice were placed on the treadmill at the speed of 3 m/min. The running speed increased every minute by 1.8 m/min, and the duration of motion was recorded until the mice showed fatigue, defined by an inability to return to the treadmill or staying on the electrical shock grids for 10 s.

### 2.4. Bone Biomechanical Testing

The right femur samples stored at −20 °C were defrosted at room temperature and then tested by a Univert biomechanical test analyzer (UV-200-01, Cellscale Biomaterials Testing, Waterloo, ON, Canada). The individual femur was placed horizontally, with the broad side of the femur facing upward on two support points with a span of 10 mm. The workstation was operated to make the probe of the tester slowly drop; the loading speed of the probe was 2 mm/min and continued to run 2 mm after the specimen broke. The original data and compression curve were obtained through calculation. The bone mechanical characteristic parameters were analyzed, including the maximum load, stiffness, energy to ultimate load, stress–strain, and breaking energy.

### 2.5. Tissue Staining

Mouse left femurs were fixed with 4% formaldehyde for 24 h, then embedded in paraffin and cut into 4 μm thick slices. Bone sections were stained by the HE kit (Servicebio, Wuhan, China) and the Masson kit (Servicebio, China). The area of the blue region in the cavity was analyzed using ImageJ v1.53.

### 2.6. Micro-CT Analysis

The left femurs were soaked in 4% paraformaldehyde over 24 h and then analyzed immediately by a micro-CT system (SkyScan1276, Bruker microCT company, Kontich, Belgium) using 60 kV voltage, 140 μA current, and 9 μm resolution ratio in the distal growth plate of femurs. After the scan was completed, the 3D images were reconstructed. Dataviewer software 1.5.6.2 was used to adjust the direction and other parameters of the scanned sample images to ensure all samples were processed under the same conditions to generate VOI images. Meanwhile, the bone density formula was constructed in CTAn software 1.17.7.2 using standard product parameters. Then, the single VOI image of each sample was imported to select 100–200 layers below the femoral growth plate to obtain bone morphometric parameters, including bone mineral density (BMD), bone volume fraction (BV/TV), trabecular thickness (Tb.Th), trabecular space (Tb.Sp) and trabecular number (Tb.N). BV/TV is the ratio of the total volume of voxels representing bone structures in the ROI to the total volume of all voxels in the region. Tb.Th is the average thickness of the trabecular bone. Tb.N is the number of intersections between bone tissue and non-bone tissue in a given length of bone. Tb.Sp is the average width of the pulp cavity between the trabeculae, indicating the porosity of the trabecular bone.

### 2.7. Determination of Calcium Content in Bone, Urine, and Stool

The left tibia of the dried mouse was placed in a container. Then, about 5 mL nitric acid and 1 mL hydrogen peroxide were added and the container was heated at 180 degrees for digestion. After the acid was volatilized to the whole volume of 1–2 mL, the volume was fixed to 50 mL with 1% dilute nitric acid. IPC-OES (Agilent company, Santa Clara, CA, USA) was used to detect the content of calcium and phosphorus ions in samples. The calcium content of each mouse’s feed, preserved urine, and feces were digested by the same machine. The IPC machine was calibrated with 50 mL, 100 μg/mL calcium standard GSB 04-2824-2011.

### 2.8. Western Blot

Jejunum and ileum tissues were lysed using RIPA buffer (Beyotime, Shanghai, China) for 30 min in ice and then centrifuged at 12,000× *g* for 15 min at 4 °C to obtain the total protein. Protein concentrations were determined by a BCA kit (Beyotime, China). Antibodies for TRPV6 (DF12784, 1:1000), S100G (DF9785, 1:1000), and Claudin-2 (AF0128, 1:1000) were purchased from Affinity (Changzhou, China). Recombinant anti-PMCA1 antibody (ab190355, 1:1000) and β-actin (ab8226, 1:5000) were obtained from Abcam (Shanghai, China), and Claudin-12 antibody (NBP1-87450,1:1000) was from Novus (Shanghai, China). Horseradish peroxidase-labeled goat anti-rabbit antibody (Beyotime, China) was used as a secondary antibody. The expression levels of TRPV6, CaBP-9k, PMCA1, CLDN2, and CLDN12 proteins were detected in the jejunum. The expression levels of CLDN2 and CLDN12 protein were detected in the ileum.

### 2.9. Statistical Analysis

All statistical analyses were performed using SPSS 26.0., and the results were shown as mean ± SEM. One-factor ANOVA followed by Duncan’s post hoc test was used to evaluate differences between groups, with different letters representing statistical significance (*p* < 0.05).

## 3. Results

### 3.1. Dietary 5% CBP Intake Significantly Increased Body Weight and Extended Exercise Exhaustion Time in Mice

**Figure 1:**
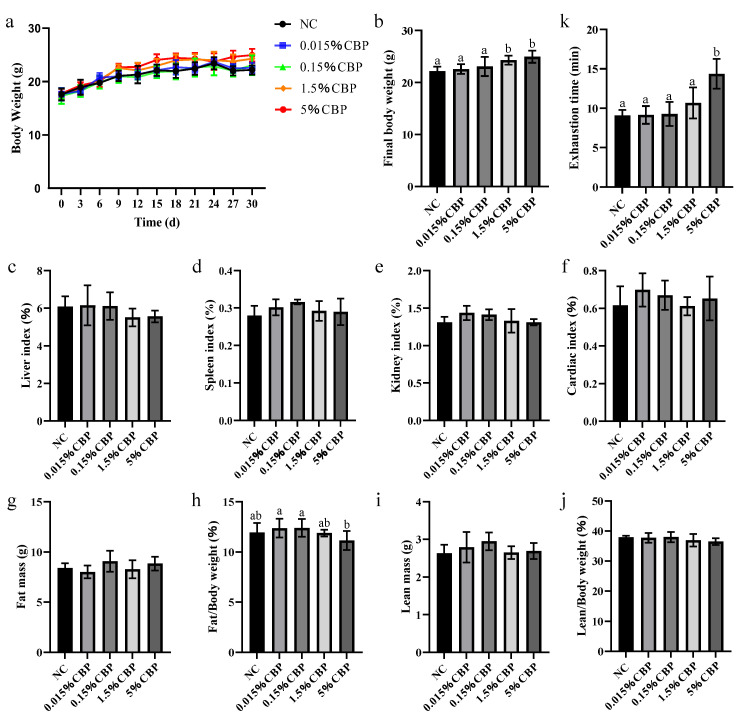
Physiological indexes and exercise indexes of mice. (**a**,**b**) Weight changes in mice. The index of liver (**c**), spleen (**d**), kidney (**e**), and heart (**f**). The fat mass (**g**), fat percentage (**h**), lean meat mass (**i**), and lean meat percentage (**j**) in mice. (**k**) Treadmill exercise exhaustion time of mice. Data are presented as mean ± SEM (*n* = 6). Statistical significance between groups is represented by different lowercase letters (*p* < 0.05).

Body weight reflects the growth and development of the body. The weight of mice was recorded every 3 days during feeding. At the start of the experiment, the weight was the same in each group, while the 1.5% and 5% CBP groups were significantly higher than that in the NC group (*p* < 0.05), which indicated that CBP intake above a certain threshold could increase the weight growth rate of mice (Figure 1a,b). The organ index is the ratio of the mass of an organ to body weight, which is used to reflect toxicological changes in the body. There was no significant difference in the organ index for the heart, liver, kidney, or spleen with increasing CBP dose, indicating that CBP in the dose range used in this study had no harmful effect on the basic physiological function of the mice (Figure 1c–f). Furthermore, CBP intake did not change the lean meat mass, lean meat percentage, or fat mass of mice but significantly reduced fat percentage at an intake of 5% CBP (*p* < 0.05) (Figure 1g–j). Treadmill experiments were conducted to assess the exhaustion time of the mice. The exhaustion time increased with increasing CBP dose, and the exhaustion time of mice in the 5% CBP dose group was significantly longer than that in the NC group (Figure 1k).

### 3.2. Dietary 5% CBP Changed Bone Morphological Indexes in Mice

**Figure 2:**
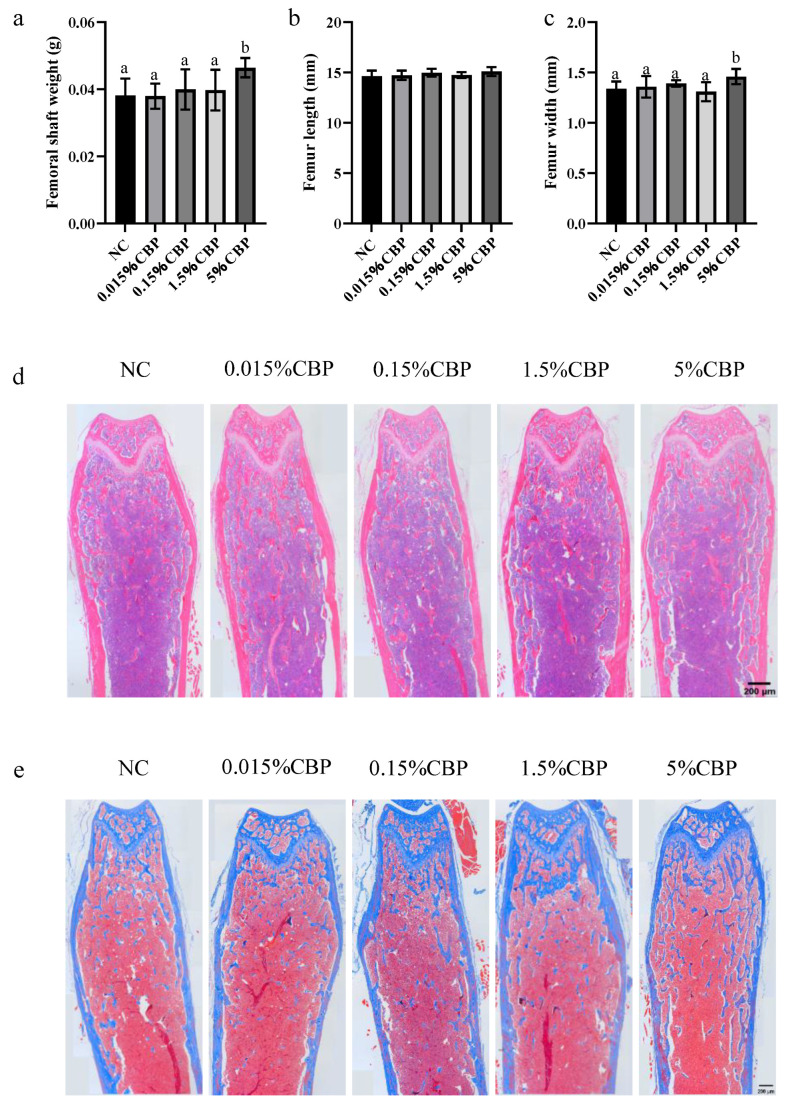
Morphological index of femur. The weight (**a**), the length (**b**), and the width (**c**) of the femur. Data are presented as mean ± SEM (*n* = 6). Statistical significance between groups is represented by different lowercase letters (*p* < 0.05). HE staining (**d**) (Scale bar = 200 µm) and Masson staining of the femur (**e**) (Scale bar = 200 µm) show bone morphology.

The weight and shape index of the femur reflect the strength of bone. In this study, the length of the femur of mice did not differ significantly in any group, but the bone weight of mice in all experimental groups increased; the bone weight of mice in the 5% CBP group was significantly higher than that in the NC group (*p* < 0.05) (Figure 2a,b). When the CBP dose was 5%, the femur width of mice was significantly increased, by 10%, compared with the NC group (*p* < 0.05) (Figure 2c), indicating that there was an improvement in strength. HE staining was used to characterize the basic bone morphology; different doses of CBP had no significant effect on the bone morphology and structure in mice (Figure 2d). Masson staining showed the distribution of collagen in bone tissue (the blue part was the collagen in the organic matter of the bone matrix). The blue area inside the bone increased with increasing CBP dose, indicating that CBP promotes the formation of organic matter in bone and makes the internal structure of bone denser (Figure 2d,e).

### 3.3. High CBP Diet Enhanced BMD and Improved Trabecular Microstructure in Mice

**Figure 3:**
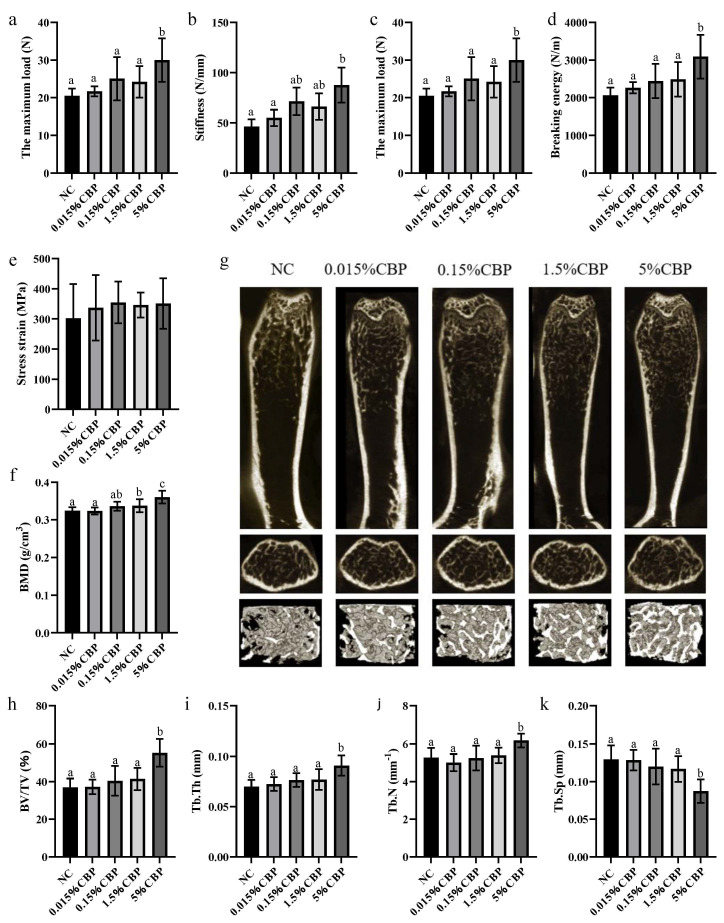
The effect of CBP on bone quantitative index. The mechanical properties of bones, including the maximum load (**a**), stiffness (**b**), fracture energy (**c**), ultimate load energy (**d**), and stress–strain (**e**). (**f**) The BMD difference between the groups. (**g**) Three-dimensional microstructure of trabecular bone of femur. Bone trabecular indicators including BV/TV (**h**), Tb.Th (**i**), Tb.N (**j**), and Tb.Sp (**k**). The data are presented as mean ± SEM (*n* = 6). Statistical significance between groups is represented by different lowercase letters (*p* < 0.05).

The index of bone mechanical properties describes the resistance of bone to stress and tension. Feeding CBP increased the maximum load, stiffness, fracture energy, and ultimate load energy of the mouse femur, among which the indexes were significantly increased in mice fed 5% CBP (*p* < 0.05) (Figure 3a–d). However, the stress–strain did not change after CBP intake. BMD is the most critical index of bone health (Figure 3e).

The result shows that BMD increased with increasing dietary CBP intake. In mice in the 5% CBP group, the BMD was increased by 10% compared with that in the NC group (Figure 3f). MicroCT econstruction technology was used to analyze the morphological structure of bone trabeculae of the distal femur of mice, and we obtained spatial parameters related to bone trabeculae for comparison so as to evaluate the effects of different doses of CBP. Meantime, the longitudinal and horizontal ROI interface and three-dimensional structure of the distal femur were obtained. With increasing CBP intake, the space within the trabecular bone gradually decreased compared with that in the NC group; the most significant change occurred in the 5% CBP group (Figure 3g).

Quantitative analysis of trabecular structural parameters showed that supplementation of the diet with CBP improved the trabecular structure in a dose-dependent manner, especially at 5% CBP. Supplementation with 5% CBP significantly increased the parameters BV/TV, Tb.Th, and Tb.N (*p* < 0.05), and remarkably decreased Tb.Sp. Compared with the NC group, BV/TV increased by 49%, Tb.Th increased by 30%, Tb.N increased by 17%, and Tb.Sp decreased by 32% in the 5% CBP group (Figure 3h–k).

### 3.4. Dietary Intake of CBP Changes Calcium Metabolism in Mice

**Figure 4:**
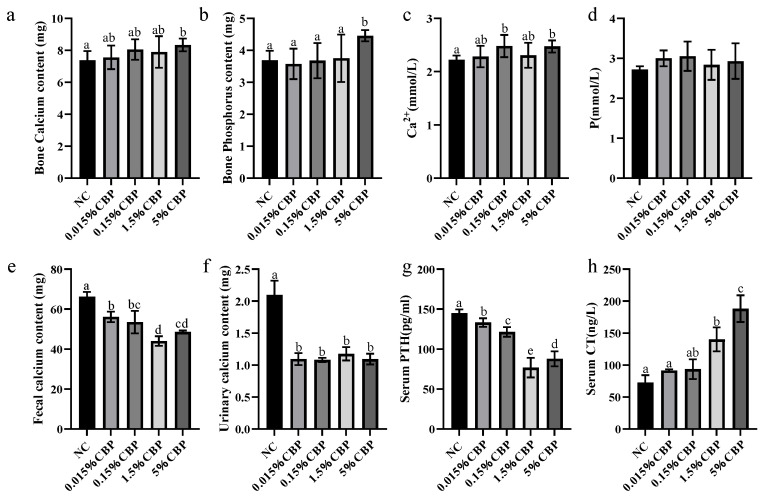
Effects of CBP on calcium metabolism in mice. (**a**,**b**) Bone calcium and bone phosphorus content (*n* = 6). (**c**,**d**) Serum calcium and phosphorus concentration (*n* = 6). (**e**,**f**) Urinary calcium and fecal calcium excretion (*n* = 3). (**g**,**h**) Serum parathyroid hormone and calcitonin concentration (*n* = 6). Data are presented as mean ± SEM. Statistical significance between groups is represented by different lowercase letters (*p* < 0.05).

Calcium is the most abundant mineral element in bone; hydroxyapatite crystals store calcium in bones. The bone calcium content of mice increased significantly in the 5% CBP group (Figure 4a). The calcium intake of mice in all groups was consistent; thus, this result indicated that the calcium absorption of mice was increased after ingesting CBP. When the CBP dose was 5%, the content of bone phosphorus in mice was significantly increased by 20% compared with the NC group (Figure 4b). Because the regulation of calcium metabolism contributes to bone mass, we evaluated changes in related minerals and hormones in the blood as well as fecal calcium of mice in each group after ingestion of CBP. We found that the serum calcium concentration of mice with intake of 0.15% and 5% CBP was significantly higher than that in the NC group (*p* < 0.05) (Figure 4c). There was no significant difference in the blood phosphorus content of mice between groups (Figure 4d).

Fluctuation in serum calcium concentration is the key factor that regulates calcium metabolism, and balance is mainly maintained through the regulation of bone calcium release and deposition, intestinal calcium absorption, and renal calcium reabsorption [11]. The excretion of urinary calcium was decreased after the intake of CBP (*p* < 0.05), indicating that CBP can promote the renal calcium reabsorption of mice, but there was no significant difference between doses. To assess the effect of CBP on intestinal calcium absorption in mice, the calcium content in mouse feces was detected. Different doses of CBP significantly decreased the amount of calcium in mouse feces (*p* < 0.05), which may be due to the promotion of intestinal calcium absorption by CBP (Figure 4e,f). The concentrations of serum PTH in each CBP intake group decreased significantly compared with the NC group (*p* < 0.05), while the increase in serum CT was CBP dose-dependent. When the CBP dose was 1.5% or higher, the CT concentration was significantly increased compared with that in the NC group (*p* < 0.05) (Figure 4g,h). The above results are consistent with the findings that after CBP ingestion by mice, serum calcium and bone calcium deposition increase; the changes in calcium metabolism after feeding CBP are conducive to bone growth.

### 3.5. High CBP Intake Increased the Expression of Proteins Related to Intestinal Calcium Absorption

**Figure 5:**
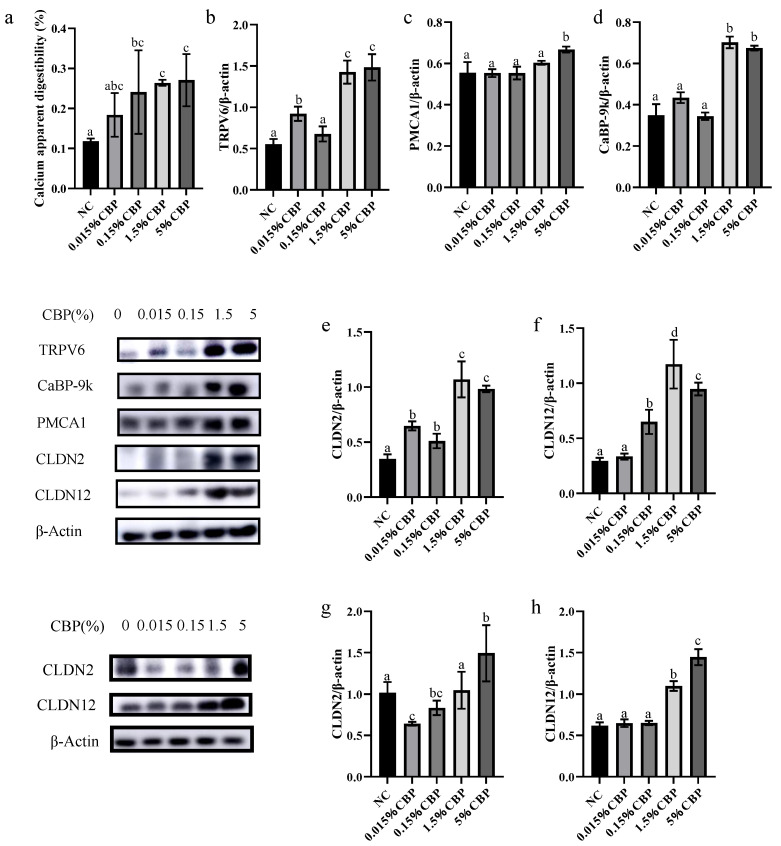
Effect of CBP on calcium absorption and related proteins in mice. (**a**) Apparent absorption rate of calcium. (**b**–**f**) TRPV6, PMCA1, CaBP-9k, CLD2, and CLD12 in jejunum, (**g**,**h**) CLD2 and CLD12 in ileum levels in the five groups. Data are presented as mean ± SEM (*n* = 3). Statistical significance between groups is represented by different lowercase letters (*p* < 0.05).

The apparent digestibility and absorption rate of calcium in the body reflects the absorption capacity of intestinal calcium. With different doses of dietary CBP, the apparent digestibility and absorption rate of calcium in mice increased to varying degrees, which was consistent with the observation of decreased excretion of fecal calcium (Figure 5a). In the jejunum of mice, both intracellular and paracellular transport of calcium play a role. The expression levels of the transcellular calcium transport proteins TRPV6, CaBP-9k, and PMCA1, and of the paracellular transport proteins CLDN2 and CLDN12, were measured in the jejunum. Doses of 1.5% and 5% CBP significantly upregulated the expression levels of TRPV6, CaBP-9k, PMCA1, CLDN2, and CLDN12 in the jejunum (*p* < 0.05) compared with the NC group. Specifically, the expression levels of TRPV6, CaBP-9k, PMCA1, CLDN2, and CLDN12 in the jejunum of the 5% CBP group were 3-, 1.4-, 2-, 3-, and 3-times those in the NC group, respectively (Figure 5b–f). Compared with other doses, the protein expression levels of CLDN2 and CLDN12 in the ileum of mice were significantly increased in the 5% CBP group (*p* < 0.05); the expression levels of CLDN2 and CLDN12 were 1.5 and 3 times those in the NC group, respectively (Figure 5g,h). The data indicate that CBP increases calcium absorption by increasing levels of the key proteins of ileal calcium paracellular transport, upregulates serum calcium concentration, and affects calcium metabolism, thereby mediating bone calcium deposition.

## 4. Discussion

Ninety percent of the bone mass of the human body is accumulated before the age of 20, and bone grows rapidly in children and adolescents. In the present study, the initially 4-week-old mice were fed for 30 days so that the feeding time essentially covered the equivalent of human childhood and adolescence. Studies have shown that 5% CBP given as a dietary supplement can significantly increase bone mineral density and serum levels of osteocalcin, growth hormone, and insulin-like growth factor-1 in young rats [14], and in 2021, our research group showed that dietary 0.015% CBP combined with milk, calcium, and vitamin D improved bone indexes in low-calcium rats. Therefore, here, four experimental feeding doses (0.015%, 0.15%, 1.5%, and 5% CBP) were applied.

In this study, the proportion of CBP was changed while the amount of total protein in the diet of the mice was kept the same. With increasing CBP dose, the weight growth rate of mice increased significantly. There was no significant difference in organ index and body composition of mice between the NC group and the CBP group, indicating that the basic physiological condition of the mice was not greatly affected by the CBP dose. We observed no significant difference in fat and lean meat mass between groups of mice. Movement of the body is coordinated by bones, muscles, and joints, and the bones, to which muscles and tendons are attached, provide support for the movement. Healthy bones are, thus, the basis of normal movement of the body, and the movement ability of the body can reflect the strength of the bones to a certain extent. Therefore, the increase in treadmill exercise ability of mice fed CBP in this study reflects the health of their bones.

The results show that a certain amount of CBP increased the weight and width of the femur in mice. The index of bone mechanical properties shows the resistance of bone to stress and tension, while the BMD directly represents the bone mass. The current study showed that a high (5%) CBP diet markedly contributes to the compression and bending resistance, rather than the elasticity, of bone. Meanwhile, 5% CBP intake significantly enhanced the BMD of the mice. Numerous previous studies have shown that the structure of bone trabeculae plays an important role in the mechanical properties of bone independently of bone density [16]. Changes in the microstructure of trabeculae, such as the number, thickness, and cross structure, affect the strength and mechanical properties of bone. The distal femur is often used to detect bone health [17]. Predictably, it was found here that the bone trabecular indexes above were significantly improved by 5% intake. Through the detection of quantitative parameters of bone health, this study comprehensively evaluated bone growth and showed that CBP is beneficial for bone growth.

The bone matrix contains 70% inorganic mineral salts, which contribute to the rigidity of bone. Calcium is the key element in bone mineral salts, and the balance of calcium metabolism in the body is of great significance for the maintenance of bone health [9]. Stable serum calcium levels ensure normal physiological activities of the body. The body produces PTH to activate osteoclasts and release bone calcium to replenish serum calcium when needed; if the serum calcium level is consistently too low, long-term effects lead to a large loss of bone calcium and bone damage. When the serum calcium level is too high, CT is produced, and osteoclast activity is inhibited. Bone absorption is reduced, and bone formation continues, which promotes the conversion of serum calcium into stored calcium in bone. Therefore, the homeostasis of calcium metabolism is a critical factor affecting bone formation [18]. Dietary calcium is the only source of calcium in the body; it is absorbed into the blood through the intestine. When the intake of calcium is the same but the absorption of intestinal calcium increases, the deposition of bone calcium will be triggered [11]. In 2021, our research group showed that dietary 0.015% CBP, in combination with milk and calcium, decreased the excretion of fecal calcium in rats. It can be seen that CBP has the potential to promote bone growth, which may be through increased intestinal calcium absorption, thereby modulating the body’s calcium metabolism.

Here, we explored whether CBP promotes bone growth by affecting calcium metabolism. It was found that the bone strength and bone calcium and phosphorus contents of mice fed a CBP-containing diet increased significantly compared with the NC group. Phosphorus is also an important component of bone; 60% of phosphorus and calcium in the body are present in bone, in a fixed ratio [18], and thus, the content of phosphorus in bone can also reflect the extent of bone calcium deposition. Meanwhile, the content of calcium and phosphorus in the blood must be maintained in proportion to maintain the balance of calcium and phosphorus metabolism [19]. After calcium ingested by the body is absorbed into the blood circulation, most of the unused calcium will be excreted in stool, and a small fraction will be excreted in urine after renal reabsorption. The amount of calcium reabsorbed by the kidney is much lower than that absorbed by the intestine, and thus, the contribution of intestinal calcium absorption to serum calcium is higher than that of renal reabsorption [20]. CBP may regulate calcium metabolism mainly by affecting intestinal calcium absorption. In this study, the decrease in calcium content in the feces of mice fed CBP was consistent with this hypothesis. The significant increase in serum calcium in the 5% CBP group may be due to the fact that CBP mainly affects the absorption of intestinal calcium, and the significant increase in serum CT observed in this study shows that the significant change in serum calcium affects the balance of calcium metabolism in the body.

The ileum accounts for >40% of bodily calcium absorption, the largest contribution to the total, and the jejunum is ranked second, accounting for about 17%. Intestinal epithelial cells absorb calcium in two main ways: energy-dependent transcellular transport and concentration-dependent paracellular transport. Absorption by the former may be saturated, while the latter is not [21]. If there is insufficient calcium intake into the body, calcium intake is dominated by transcellular transport in the duodenum. In the case of sufficient calcium intake, paracellular transport in the ileum dominates (while transcellular transport of calcium in the ileum is silent). Both modes of calcium transport occur in the jejunum. In this study, we found that the expression levels of TRPV6, CaBP-9k, PMCA1, CLDN2, and CLDN12 were all increased in the jejunum by 5% CBP. The expression levels of CLDN2 and CLDN12 in the ileum were also increased in the 5% CBP group. These findings were consistent with the characteristic calcium absorption modes in each intestinal segment, indicating that CBP had a promoting effect on intestinal epithelial calcium absorption. Because the calcium absorption rate of the ileum is significantly higher than that of the jejunum, and the jejunum contains more intestinal endocrine calcium, the calcium absorption of the ileum contributes more to overall serum calcium change. We thus speculate that CBP mainly mediates bone growth by promoting the expression of key proteins in the ileum.

However, we believe that it is useful to explore the harm of high doses of CBP to the human body, and whether it contains bound calcium itself is also worth studying. This study explored the function of CBP in promoting calcium absorption in vivo, suggesting that CBP may be used in combination with calcium as a supplement to achieve better bone-promoting effects in future product applications. In our experiments, CBP may be more appropriate to be used to increase peak bone mass in children and adolescents. Its role in older adults with advanced bone loss or osteoporosis is unclear.

## 5. Conclusions

**Figure 6:**
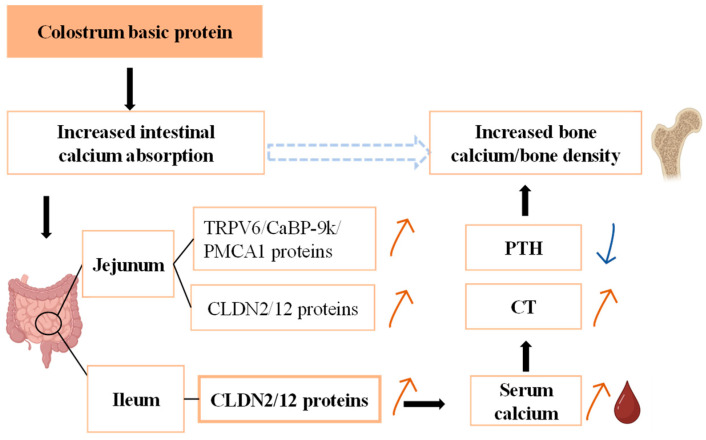
Schematic diagram of CBP mediating bone growth. CBP upregulates the expression of intestinal paracellular transport protein, promotes calcium absorption, and increases serum calcium concentration, thereby mediating the regulation of calcium metabolism in the body and promoting bone calcium deposition. The black arrow indicates that there is a regulatory relationship. The red arrow indicates that it is upregulated, and the blue arrow indicates that it is downregulated.

Overall, this study demonstrates that dietary CBP upregulates the expression of intestinal paracellular calcium transport proteins, promotes calcium absorption, and increases the serum calcium concentration, thereby mediating the regulation of calcium metabolism in the body and promoting bone calcium deposition in mice (Figure 6), and intake of 5% CBP had the best effect.

## Figures and Tables

**Table 1:** Main nutrients of CBP.

Composition	Content (%)
Protein	≥80%
1–30 kDa molecular weight protein/peptide	≥50%
Water	≤7%
Ash	≤3%

**Table 2:** Composition of basal diet and proportion of nutrient components.

Composition	Content (%)
Total protein	20.0
Starch	39.7
Cystine	0.3
Maltodextrin	13.2
Cane sugar	10.0
Cellulose	5.0
Soybean oil	7.0
TBHQ antioxidant	0.0014
Mixed salt	3.5
Mixed vitamin	1.0
Choline tartrate	0.25

## Data Availability

Data are contained within the article.
